# Optimized Enoxolone-Loaded Microsponges for Drug Delivery: A Design of Experiments Approach

**DOI:** 10.3390/ph18070938

**Published:** 2025-06-21

**Authors:** Juste Baranauskaite, Sedef Sefer, Augusta Zevzikoviene, Andrejus Zevzikovas, Liudas Ivanauskas

**Affiliations:** 1 Department of Pharmaceutical Technology, Faculty of Pharmacy, Yeditepe University, Kayisdagi Cad., Atasehir, 34755 Istanbul, Türkiye; juste.ortasoz@yeditepe.edu.tr (J.B.); sedef.sefer.std@yeditepe.edu.tr (S.S.); 2 Department of Analytical and Toxicological Chemistry, Lithuanian University of Health Sciences, Mickeviciaus Str. 9, 44307 Kaunas, Lithuania; andrejus.zevzikovas@lsmu.lt (A.Z.); liudas.ivanauskas@lsmu.lt (L.I.)

**Keywords:** microsponges, enoxolone, chronic periodontitis

## Abstract

Enoxolon is widely recognized for its pharmacological potential, exhibiting antioxidant, anti-inflammatory, anticancer, and antiviral properties. **Objectives**: This study aimed to develop an enhanced formulation of enoxolone-loaded microsponges as a novel drug delivery system. A design of experiments (DoE) approach was employed for the optimization process. **Methods**: The microsponges were produced using the *quasi*-emulsion technique. The selected formulation was evaluated for yield, particle size, and entrapment efficiency. Furthermore, the microsponges were incorporated into a 1% MC solution matrix, and in vitro release studies were performed to assess their drug delivery performance. **Results**: The optimal formulation was determined through the DoE methodology, which involved varying the concentrations of methylcellulose (MC) (0.55–1.87%, *w*/*w*), polyvinyl alcohol (PVA) (0.5–1%, *w*/*w*), and Tween 80 (TW80) (1.5–2.5%, *w*/*w*). The results showed a particle size of 142.8 ± 10.02 µm and an entrapment efficiency of 80.3 ± 1.99%. When comparing the optimized microsponge formulation to pure enoxolon, a 1.29 times higher release rate was observed (*p* ≤ 0.05). **Conclusions**: Following optimizationand physicochemical characterization studies were conducted to further assess the formulation. These findings suggest that microsponge-based delivery systems hold considerable potential as an alternative platform for the topical treatment of chronic periodontitis.

## 1. Introduction

Glycyrrhizinic acid (enoxolone), often referred to as glycyrrhizin, is a triterpenoid saponin extracted from the roots and rhizomes of *Glycyrrhiza glabra* (licorice) [1]. The dried root extract generally contains 4% to 25% of this active compound, along with other bioactive constituents such as polyphenols, saponins, and triterpenes, including glycyrrhetinic acid (enoxolone). Enoxolon is widely recognized for its pharmacological potential, exhibiting antioxidant, anti-inflammatory, anticancer, and antiviral properties. One of glycyrrhizin’s notable effects is its interaction with high-mobility group box 1 (HMGB1), where it inhibits the protein’s function [2]. Glycyrrhizinic acid (enoxolone) could be beneficial for use in the treatment of chronic periodontitis (CP), a bacterial-driven inflammatory disease affecting the gums and other periodontal structures [3]. Several factors contribute to its development, including bacterial infections, environmental influences, and host immune responses. A hallmark of CP is inflammation, primarily triggered by the *Gram-negative* bacterium *Porphyromonas gingivalis* (Porphyromonadaceae) [4]. Under inflammatory conditions, such as those triggered by LPS or TNF-α, HMGB1 can be actively released from cells and function as a pro-inflammatory cytokine. Studies suggest that extracellular HMGB1 plays a critical role in inflammatory diseases, including CP. Researchers have proposed that targeting HMGB1 with neutralizing antibodies could alleviate CP symptoms, making it a promising therapeutic target [5].

Traditional oral drug formulations generally release active pharmaceutical ingredients into the gastrointestinal tract, where absorption is influenced by the drug’s physicochemical properties. However, many commercially available immediate-release drugs experience substantial pre-systemic metabolism, leading to poor bioavailability or premature elimination before the next scheduled dose [6]. This inefficiency may result in suboptimal therapeutic outcomes. To address these challenges, controlled drug delivery systems have been developed to reduce dosing frequency, bypass gastric degradation, achieve targeted drug release, enhance therapeutic efficacy, and improve patient safety by minimizing fluctuations in drug concentration. The field of drug delivery is rapidly evolving, with increasing competition driving innovations to improve treatment efficiency and cost-effectiveness [7].

Among these advancements, microparticulate drug carriers have gained attention for their ability to regulate drug release through rate-controlled or site-specific mechanisms—or a combination of both—while maintaining formulation versatility. These multiparticulate systems ensure more uniform distribution throughout the absorption site, leading to better drug uptake. Additionally, microparticles offer promising potential for chronotherapeutic drug delivery. Various types of microparticulate systems have been explored, including microspheres, microbeads, microcapsules, microballoons, and microsponges [8].

The Microsponge Delivery System (MDS) is a patented polymeric system composed of porous microspheres. These sponge-like particles contain interconnected voids within a non-collapsible structure, creating a highly porous surface that enhances drug diffusion. Microsponge sizes typically range from 5 to 300 µm. The MDS has been widely used in topical formulations, improving drug penetration by maintaining active ingredients at their highest thermodynamic activity in an optimized carrier system [9]. Microsponges also provide a cost-effective means of encapsulating various liquid or soluble drugs [10]. For successful incorporation into microsponges, active ingredients must either be completely miscible in the monomer or made compatible using a small quantity of a water-immiscible solvent. Due to their ease of production, high-drug-loading-potential microsponges continue to be widely investigated as advanced drug carriers [11].

Although enoxolone has a long history of traditional use and is known for its diverse phytochemical properties, to the best of our knowledge, no prior studies have focused on its formulation in a topical delivery system. Salari et al. presented in their studies that enoxolone is effective against periodontopathogenic and capnophilic bacteria [12]. Moreover, Baranauskaite-Ortasoz et al. produced PVP-based orodispersible films (ODFs) containing enoxolone via electrospinning and solvent casting methods to compare them in terms of their credibility for the orodispersible delivery of enoxolone through a variety of in vitro and physical investigations [13]. This study presents the first attempt to develop a microsponge-based gel formulation of enoxolone for topical application. Microsponges are particularly well suited for encapsulating herbal extracts, as they can significantly reduce drug-induced irritation without compromising therapeutic efficacy [14]. One of the primary challenges in utilizing natural extracts, including their administration via the oral mucosa, is their limited bioavailability [15]. Incorporating enoxolone into a microsponge delivery system offers a promising strategy for overcoming these limitations.

Enoxolone’s therapeutic potential is significantly limited by its pronounced hydrophobicity and poor bioavailability. To overcome these challenges, polymer-based carrier systems offer a promising strategy for enhancing drug delivery. The present study aimed to formulate and optimize enoxolone-loaded microsponges as an alternative delivery platform. A Design of Experiments (DoE) approach was employed to systematically optimize the formulation parameters. The resulting microsponges were subjected to comprehensive physicochemical characterization, including assessments of production yield, drug loading, and entrapment efficiency. Subsequently, the optimized microsponges were incorporated into a hydrogel matrix, and in vitro release studies were conducted to investigate their drug release profiles.

## 2. Results and Discussion

### 2.1. Preparation of Enoxolon Microsponges

Enoxolon microsponges were synthesized using the *quasi*-emulsion technique, where the interaction between the organic solvent and water triggered polymer precipitation and the evaporation of dichloromethane during stirring, resulting in the formation of porous microsponges [16]. Preliminary experiments showed that the production yield of the microsponge formulations was influenced by the polymer concentration in both the aqueous and organic phases. Higher concentrations of polymer enhanced the product yield and encapsulation efficiency [17].

Furthermore, the stirring speed was found to play a critical role in determining the particle size. Faster stirring rates produced finer particles, while slower speeds led to aggregation. Based on these observations, a stirring speed of 15,000 rpm was chosen for subsequent experiments. Statistical optimization of the microsponge formulations was carried out by varying the concentrations of enoxolon, MC, PVA, and Tween 80 within the following ranges: 0.04%, 0.5–1.87%, 0.5–1.0%, and 1.5–2.5%, respectively.

### 2.2. Statistical Analysis Using D-Optimal Mixture Design

The optimal microsponge formulation, aimed at maximizing encapsulation efficiency and minimizing particle size, was determined through an experimental mixture design. Sixteen formulations were prepared with varying concentrations of methyl cellulose (MC), polyvinyl alcohol (PVA), and Tween 80. The formulations were then assessed for mean particle size and encapsulation efficiency (Table 1). The primary goal was to develop a balanced formulation that maximizes encapsulation efficiency while minimizing particle size. The key results are summarized in Table 2, and the fitting models along with statistical parameters are detailed in Table 2.

The encapsulation efficiency ranged from 58.3 ± 1.04% to 81.02 ± 1.99%, demonstrating the significant impact of MC, PVA, and Tween 80 concentrations on microsponge formation. When the MC and PVA concentrations were reduced to 0.55% and 0.5%, respectively, and Tween 80 was increased to 2%, the encapsulation efficiency improved. This may be attributed to increased viscosity at higher polymer concentrations, leading to a more rigid polymeric shell that hinders drug diffusion, thereby lowering encapsulation efficiency [18]. Additionally, drug loading efficiency remained below 100% across all formulations, likely due to partial drug dissolution in the solvent or aqueous phase. Higher drug-loading efficiencies were observed at lower drug-to-polymer ratios. When higher PVA concentrations were used in formulations with increased polymer-to-drug ratios, the viscosity of the dispersed phase slightly increased. Upon solvent diffusion, most of the dispersed phase solidified into microsponges, forming distinct particles. This suggests that greater polymer availability per drug unit contributed to improved drug retention [18]. In agreement with our findings, Martinez-Borrajo et al. found out that higher EE was found with greater polymer concentrations [19]. Similar results were found by other authors [20]. For optimal performance, microsponge particle sizes should range from 5 to 300 µm. The study revealed that the mean particle size varied from 141.8 ± 6.14 µm to 303.4 ± 12.2 µm, significantly influenced by the MC, PVA, and Tween 80 concentrations. An increase in the drug-to-polymer ratio led to larger microsponges due to the formation of thicker polymer walls. Similarly, higher PVA concentrations contributed to increased particle size by increasing the viscosity and stabilizer concentration. These findings align with previous studies, which reported that larger emulsion droplets resulted in increased microsponge size [21,22,23].

In conclusion, the optimal microsponge formulation was achieved with 0.1% enoxolon, 1.09% MC, 0.67% PVA, and 2.14% Tween 80, yielding uniform, spherical particles. Further numerical optimization using the desirability function confirmed these parameters (Table 3). The experimental results closely matched the predicted values, with no statistically significant differences (*p* > 0.05), validating the optimization approach.

### 2.3. The Production Yield

**Figure 1:**
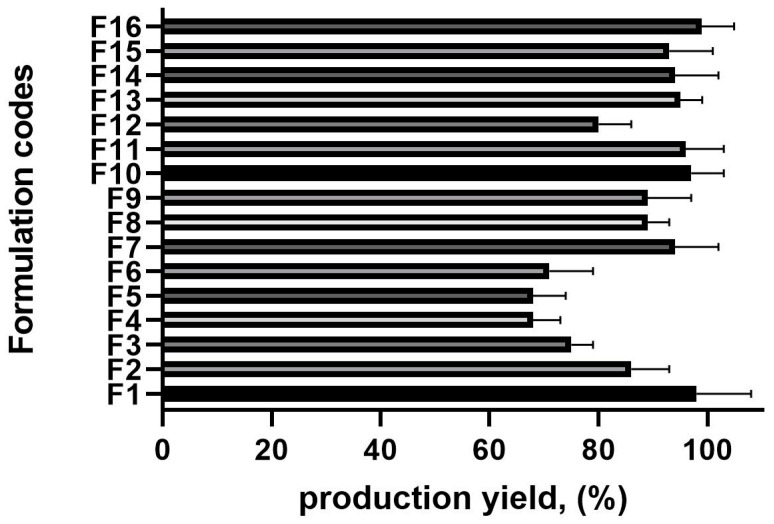
The production yield results of the microsponge formulations, n = 6 (the composition of the formulation is shown in Table 1).

The production yield across all batches ranged from 68.36 ± 0.98% to 98.58 ± 0.64%, significantly influenced by the drug-to-polymer ratio and polyvinyl alcohol (PVA) concentration (Figure 1). An increase in the drug-to-polymer ratio led to a higher production yield. For instance, at a 1:15 drug-to-polymer ratio (F4), the production yield was relatively low (68.3%), whereas at a 1:22 ratio (F16), it reached 98.9%.

Similarly, the PVA concentration played a crucial role in yield optimization. When the PVA was at its lowest concentration (0.5%, F6), the production yield was 70.01%. However, as the PVA concentration increased (from 0.65% to 1%), the production yield improved. This can be attributed to the reduced diffusion rate of dichloromethane from the polymer solution to the aqueous phase at higher drug-to-polymer ratios, allowing more time for droplet formation and, consequently, increasing yield [21].

### 2.4. In Vitro Drug Release Study

**Figure 2:**
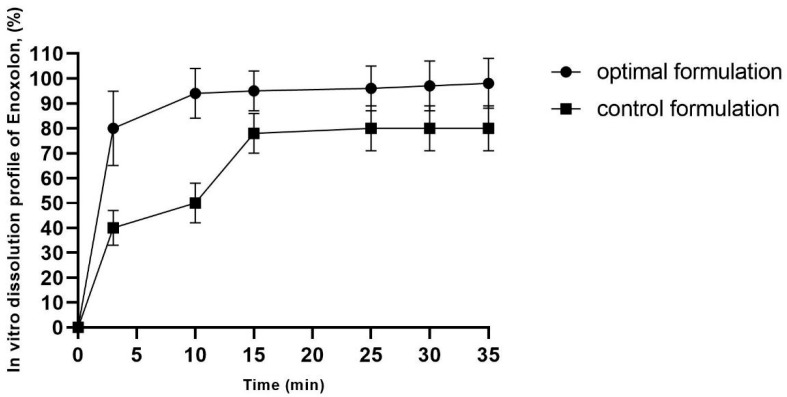
In vitro enoxolon dissolution profile of optimal enoxolon microsponge formulation. The optimal enoxolon microsponge formulation contains 0.1% of enoxolon, 1.094% of methyl cellulose, 0.67% of polyvinyl alcohol, and 2.14% of Tween 80, and control formulation contains 0.1% enoxolon and 1% of methylcellulose.

The drug release profiles of the optimal enoxolon microsponge (the optimal enoxolon microsponge formulation contains 0.1% of enoxolon, 1.094% of methyl cellulose, 0.67% of polyvinyl alcohol, and 2.14% of Tween 80, and is dispersed in 1% MC solution) and control formulations (0.1% of enoxolon dispersed in 1% MC solution) are depicted in Figure 2. The release mechanism can be attributed to the porous structure of the microsponges, which facilitates the penetration of the dissolution medium and enhances access to the entrapped drug molecules [24].

An initial burst release was observed, with 90% of the drug released within the first minute. This effect became more pronounced after 7 min. The burst release can be explained by the intrinsic porosity of the microsponges, as the interconnected pores provide channels for rapid drug diffusion [25]. Additionally, 80% of enoxolon was released within 3 min from the optimized formulation, indicating the microsponges’ ability to release the drug efficiently (Figure 2).

The initial rapid drug release can be attributed to surface-adsorbed drug molecules, which dissolve quickly to establish equilibrium with the dissolved fraction. According to the literature, controlled drug release can be achieved through the uniform mixing of the drug and polymer in the organic phase, ensuring a homogeneous formulation [26]. Burst release formulations in the local delivery of active substances offer some advantages in terms of delivering high drug concentrations directly to the infection site, ensuring rapid antimicrobial action. Their ability to enhance efficacy, improve patient compliance, and reduce systemic side effects makes them a valuable tool in periodontal therapy [27,28].

Finally, when comparing the optimized microsponge formulation to pure enoxolon, a significantly higher release rate was observed (*p* ≤ 0.05). This could be due to the impact of the porous structure and the physical properties of the microsponges, which enhance drug diffusion and release efficiency.

## 3. Materials and Methods

### 3.1. Materials

Glycyrrhizinic acid was acquired from Rapharm (Athens, Greece). Methyl cellulose was supplied by Evonik GmbH (Essen, Germany). Polyvinyl alcohol (M_w_ 31,000–50,000, 87–89% hydrolyzed), Tween 80, dichloromethane, and methanol were purchased from Sigma-Aldrich (Darmstadt, Germany).

### 3.2. Optimization of Microsponge Composition Using D-Optimal Mixture Design

The D-optimal mixture design was used to optimize the composition of the microsponge formulation by using Design Expert Software (version 13.0.2.0, State-Ease Inc., Minneapolis, MN, USA). The experiment was carried out using the four components as independent variables: enoxolon, MC, PVA, and TW80 were set within ranges of 0.1%, 0.55–1.87%, 0.5–1.0%, and 1.5–2.5%, respectively. The ranges of polymers were chosen according to previous studies [29]. The mean particle size and encapsulation efficiency were evaluated to determine the optimal microsponge formulation with excellent physical–mechanical properties. A total of 16 runs were generated by the software and the design matrix is shown in Table 2. The results of the analysis were expressed as a mean value of 3 measurements.

### 3.3. Preparation of Microsponges

Microsponges were fabricated using a *quasi*-emulsion technique. The process began with the preparation of an organic phase by dissolving the required amount of methyl cellulose in the required amount of dichloromethane, as detailed in Table 1. To create a water-in-oil (*w*/*o*) emulsion, a porogenic solution was gradually introduced into this organic phase. The porogenic solution, prepared in advance, consisted of an aqueous solution of Tween 80 (0.5–1.8% *w*/*v*) to achieve a final dispersion concentration of 1.5–2.5% *w*/*v*. Glycyrrhizinic acid was incorporated during the final stage under continuous magnetic stirring at 1500 rpm for 1 h. Subsequently, the *w*/*o* emulsion was dispersed into an aqueous polyvinyl alcohol (PVA) solution, forming a water-in-oil-in-water (*w*/*o*/*w*) double emulsion. The system was continuously stirred for 24 h to ensure the complete evaporation of the organic solvent. The resulting microspheres were then collected via centrifugation at 15,000 rpm for 10 min, followed by drying at 40 °C in a hot air oven. Finally, the dried microsponges were stored in a desiccator for subsequent analysis [16,29]. Each formulation was prepared in triplicate.

### 3.4. Particle Size Analysis

The particle size distribution was analyzed using a technique with a Zetasizer Nano ZS (Malvern Instruments, Malvern, UK). A 1% (*w*/*v*) aqueous dispersion of the microsponges was inserted into a laser diffraction instrument. The Mastersizer 3000 software was used to record the average particle diameter and span factor. The span factor typically includes the width of the droplet size distribution. The results of the analysis were expressed as the mean value of 10 measurements. It may be determined using the equation here: [29]

(1) $$Span=\frac{d_{90-d_{10}}}{d_{50}}$$

### 3.5. Determination of Yield, Drug Content, and Entrapment Efficiency

The product yield was determined by calculating the ratio of the actual weight of the microsponges to the total weight of the drug and polymers used in the formulation, expressed as a percentage. To evaluate the drug content and entrapment efficiency, 10 mg of microsponges was placed in a 5 mL volumetric flask containing 200 µL of methanol, facilitating the breakdown of microsponges and the extraction of enoxolone. The mixture underwent ultrasonication for 10 min, followed by filtration to remove particulate matter. The drug content and entrapment efficiency were quantified using High-Performance Liquid Chromatography (HPLC) using the Waters 2695 HPLC system (Waters, Milford, CT, USA) equipped with a Waters 996 PDA detector. The results of the analysis were expressed as a mean value of 6 measurements. The calculations for drug content and entrapment efficiency were performed using the following equation [21,30]:

(2) $$Drug\;Content\;\left(\%\right)=\frac{Practical\;drug\;content\;in\;microsponges}{weight\;of\;microsponges}\times 100,$$

(3) $$Entrapment\;efficiency\;\left(\%\right)=\frac{Practical\;drug\;content\;in\;microsponges}{theoretical\;drug\;content}\times 100,\;$$

### 3.6. In Vitro Drug Release Studies

In vitro dissolution studies of the MC–microsponges and enoxolon were conducted using a Type II dissolution apparatus. The MC–enoxolon microsponges and enoxolone were dispersed in a 1% MC solution and suspended in 900 mL of pH 6.8 phosphate buffer as the dissolution medium. The system was maintained at 37 °C ± 0.5 °C, with the paddle rotating at 50 rpm. Samples were withdrawn at specified time intervals over a 35 min period to evaluate the drug release profile and release mechanism. The drug concentration in the collected samples was determined using High-Performance Liquid Chromatography (HPLC) using the Waters 2695 HPLC system (Waters, Milford, CT, USA) equipped with a Waters 996 PDA detector. To maintain the volume of the dissolution medium at 900 mL, an equal volume of fresh buffer was added after each sample collection. The evaluation of dissolution profiles was carried out in triplicate [31].

### 3.7. High-Performance Liquid Chromatography Analysis of Enoxolon

HPLC analysis was performed using the Waters 2695 HPLC system (Waters, Milford, CT, USA) equipped with a Waters 996 PDA detector. The data was collected and analyzed using a PC and the Empower2 chromatographic manager system (Waters Corporation, Milford, USA). For the determination of enoxolon ACE C18, 250 × 4.6 mm, 5 µm column (Advanced Chromatography Technologies, Aberdeen, Scotland) was used at 30 °C. The two elution solvents were exchanged: the solvent A ((0.1% *v*/*v*) trifluoracetic acid in water) and the solvent B (acetonitrile). The following linear gradient elution profile was used: 95% A/5% B–0–1 min, 70% A/30% B–10 min, 50% A/50% B–15 min, 0% A/10% B–20–28 min, and 95% A/5% B–28–35 min. The flow rate was 1 mL/min and the injection volume was 10 μL. The effluent was determined at a wavelength of 254 nm. The retention time was 18.283 min.

In order to validate the HPLC method, the international guidelines on the analytical techniques for pharmaceutical quality control were followed [32]. In order to validate the method, the linearity, limits of detection (LOD) and quantitation (LOQ), accuracy, precision, specificity, selectivity, and stability were evaluated [33]. The LOD and LOQ values were 0.095 mg/mL and 0.420 mg/mL, respectively. The analytical method displayed linearity between 0.480 and 492 µg/mL (y = 8.20⋅103X + 5.19⋅103); with R^2^ = 0.997.

### 3.8. Statistical Analysis of Data

The production yield, drug content, and entrapment efficiency of the various formulations were statistically analyzed using the Design of Experiments (DoE) approach, with the data processed using Design Expert software, Version 13.0. The raw data was assessed using ANOVA statistical testing (specifically one-way analysis of variance) and Tukey’s multiple comparison test. For this purpose, a software package was utilized (Prism v. 5.04, GraphPad Software Inc., La Jolla, CA, USA), with statistical significance being defined as *p* < 0.05.

## 4. Conclusions

The therapeutic utility of enoxolone is considerably constrained by its inherent hydrophobicity and limited bioavailability. To address these limitations, microsponge-based carrier systems represent a promising strategy for topical drug delivery, enabling rapid release and improved therapeutic efficacy. In this study, enoxolone-loaded microsponges were synthesized via the *quasi*-emulsion solvent diffusion method. A Design of Experiments (DoE) framework was implemented to systematically optimize formulation variables. The developed microsponges underwent extensive physicochemical characterization, including evaluations of production yield, drug loading capacity, and entrapment efficiency. In vitro characterization results indicated that the optimized formulation was suitable for local administration. Moreover, comparison of the enoxolone-loaded microsponge solutions with a solution containing pure enoxolone demonstrated a significantly enhanced drug release profile. These findings suggest that microsponge-based delivery systems hold considerable potential as an alternative platform for the topical treatment of chronic periodontitis. Lastly, preformulation studies have been carried out, and it could be beneficial to continue the research on in vivo studies to confirm the suitability of the newly developed formulation for the treatment of chronic periodontitis while using rapid release and a high encapsulated amount of microsponge formulations.

## Figures and Tables

**Table 1:** Composition and observed responses from randomized runs in the D-optimal mixture design.

Formulation Codes	Enoxolon (%; X_1_)	MC (%; X_2_)	PVA (%; X_3_)	TW 80 (%; X_4_)	EE (%; Y_1_)	Size (µm; Y_2_)
F1	0.1	1.53	0.87	1.50	63.8 ± 1.83	228.3 ± 6.5
F2	0.1	1.59	0.62	1.68	68.02 ± 2.09	258.0 ± 10.8
F3	0.1	1.18	0.50	2.22	76.3 ± 1.26	230.8 ± 6.1
F4	0.1	0.95	0.50	2.45	74.8 ± 0.54	228.1 ± 14.1
F5	0.1	1.46	0.50	1.93	74.6 ± 1.69	195.5 ± 9.3
F6	0.1	1.87	0.50	1.54	59.3 ± 1.84	303.4 ± 12.2
F7	0.1	0.55	0.85	2.50	75.02 ± 2.24	203.1 ± 10.4
F8	0.1	1.22	0.68	1.99	75.7 ± 1.37	154.7 ± 12.1
F9	0.1	0.84	0.79	2.28	78.21 ± 1.19	216.7 ± 14.3
F10	0.1	1.22	0.69	1.99	81.02 ± 1.99	142.2 ± 7.28
F11	0.1	0.89	1.00	2.01	78.69 ± 2.24	141.8 ± 6.14
F12	0.1	0.89	1.00	2.01	78.03 ± 1.87	143.5 ± 9.24
F13	0.1	1.22	0.68	1.99	80.3 ± 1.99	142.8 ± 10.02
F14	0.1	1.53	0.87	1.50	58.3 ± 1.04	228.7 ± 9.88
F15	0.1	0.55	0.85	2.50	74.2 ± 1.66	203.4 ± 3.73
F16	0.1	1.19	1.00	1.70	70.3 ± 1.03	148.3 ± 9.2

MC = methylcellulose; TW 80 = Tween 80; and PVA = polyvinyl alcohol. Data are presented as mean (n = 3) ± SD.

**Table 2:** Results of regression analysis for the measured responses.

Response	Model	R^2^	R^2^ Predicted	R^2^ Adjusted	Std Dev.
Particle size	Quadratic	0.9234	0.8234	0.9245	21.45
Encapsulation efficiency	Quadratic	0.9901	0.9234	0.9678	10.12

**Table 3:** Numerical optimization of ingredient amounts for microsponges using desirability function.

	Independent Variables		
Ingredients	Amount Levels (%)	Predicted Optimal Amount (%)	
Enoxolon	0.1	0.1	
MC	0.5–1.9	1.09	
PVA	0.5–1.0	0.67	
TW 80	1.5–2.5	2.14	
	**Response variables**		
	**Criteria**	**Predicted**	**Obtained**
Particle size (µm)	Minimize	141.8 ± 18.4	142.34 ± 8.2
Encapsulation efficiency (%)	Maximize	79.50 ± 1.9	78.96 ± 2.4

Desirability 0.966.

## Data Availability

Data is contained within the article.
